# Association Study of Anticitrullinated Peptide Antibody Status with Clinical Manifestations and SNPs in Patients Affected with Rheumatoid Arthritis: A Pilot Study

**DOI:** 10.1155/2022/2744762

**Published:** 2022-05-11

**Authors:** Argul Issilbayeva, Bayan Ainabekova, Sanzhar Zhetkenev, Assel Meiramova, Zhanar Akhmetova, Karlygash Karina, Samat Kozhakhmetov, Madiyar Nurgaziyev, Laura Chulenbayeva, Dimitri Poddighe, Jeannette Kunz, Almagul Kushugulova

**Affiliations:** ^1^Laboratory of Human Microbiome and Longevity, Center for Life Sciences, National Laboratory Astana, Nazarbayev University, Nur-Sultan, Kazakhstan; ^2^NJSC Medical University Astana, Department of Internal Medicine with the Course of Gastroenterology, Endocrinology and Pulmonology, Nur-Sultan, Kazakhstan; ^3^Department of Medicine, Nazarbayev University School of Medicine (NUSOM), Nur-Sultan, Kazakhstan; ^4^Department of Pediatrics, National Research Center for Mother and Child Health, University Medical Center, Nur-Sultan, Kazakhstan

## Abstract

**Introduction:**

Rheumatoid arthritis (RA) is an autoimmune disease of unknown etiology that leads to disability due to articular and extra-articular damage. RA prevalence is variable. The disease is most common among females with a 3 : 1 ratio. The interaction of environmental and host factors contributes to RA development. Currently, the genome-wide association studies (GWAS) give the opportunity to uncover the RA genetic background. Anticitrullinated peptide antibody (ACPA) is a highly specific RA antibody, associated with poor prognosis and severe course of RA, and regulated by numerous genes. Our study is aimed at investigating whether there are any clinical and genetic aspects correlate with ACPA presence in Kazakhstani patients with RA. Indeed, the available studies on this subject are focused on Caucasian and East Asian populations (mainly Japanese and Chinese), and there are scarce data from Central Asia.

**Methods:**

Our study included 70 RA patients. Patients' blood samples were collected and genotyped for 14 SNPs by real-time polymerase chain reaction (RT-PCR). General examination, anamnestic, and clinical and laboratory data collection were carried out. Statistical analysis was performed using R statistics. *Results and Conclusion*. Our study revealed a significant association of ACPA positivity with Fc receptor-like 3 (FCRL3) and ACPA negativity with signal transducer and activator of transcription 4 (STAT4) genes, but not with T cell activation Rho GTPase activating protein (TAGAP). In addition, ACPA positivity was associated with radiographic progression, rheumatoid factor (RF), erythrocyte sedimentation rate (ESR), age of RA onset, the patient global assessment, body mass index (BMI), and Gamma globulin.

**Conclusion:**

Remained 11 earlier identified significantly associated in Caucasian and Asian population SNPs were not replicated in our cohort. Further studies on larger cohorts are needed to confirm our findings with higher confidence levels and stronger statistical power.

## 1. Introduction

Rheumatoid arthritis (RA) is an autoimmune disease of unknown etiology that leads to disability due to articular and extra-articular damage. RA prevalence is variable. [1]. The disease is most common among females, making a ratio of 3 : 1 [2]. The interaction of environmental and host factors contributes to RA development [3]. The genetic factor is leading one [4, 5]. Currently, the genome-wide association studies (GWAS) give the opportunity to uncover genetic background of RA. The majority of pathogenetically crucial in RA development HLA and non-HLA genes have been revealed [6–10]. HLA DRB genes certainly occupied the major niche in RA etiopathogenesis [11]. The large-scale study by Okada et al. has presented more than 100 non-HLA RA gene loci, confirming their pivotal role in the RA development [10]. Among these genes, the most key and widely studied ones can be distinguished; moreover, a specific role in the RA pathogenesis is assigned to each of them. Accordingly, PADI2 (peptidyl arginine deiminase 2) promotes citrullination of proteins resulting in the conversion of arginine into citrullines [12]. Several risk factors affect T cell activation of function. These include CD28 and CD40, which affect T cell proliferation and proinflammatory cytokine synthesis [13, 14]. The CTLA4 (cytotoxic T-lymphocyte associated protein 4) sends the inhibitory signal to T cells and provides its regulation [15], STAT4 (signal transducer and activator of transcription 4) provides phosphorylation in response to proinflammatory cytokines and activates the transcription process under the interleukin-12 control [16], TAGAP (T cell activation Rho GTPase activating protein) regulates T-cell activation effecting its cytoskeleton [17], COG6 (component of oligomeric Golgi complex 6) provides a Golgi apparatus functioning [18], TRAF1 (tumor necrosis factor receptor-associated factor-1) encoding the certain protein of TNF superfamily mediates the antiapoptotic signals coming from TNF receptors [19], ETS1 (proto-oncogene 1, transcription factor) encodes a protein responsible of a large number of gene expression leading to stem cells and tumor process development [20], FCRL3 (Fc receptor like-3) encodes a protein that is an Fc receptor-like glycoprotein capable of influencing the activation of immunoreceptor-tyrosine [21], LBH (limb bud and heart development) regulates WNT signaling pathway [22], and the roles of LINC01104 (long intergenic nonprotein coding RNA 1104) and other genes are being investigated. Further studies aimed at validating early identified SNPs in different populations demonstrated significant variabilities in genetic predisposition to RA in various ethnicities [8, 23–29].

Approximately two-thirds of RA patients produce anticitrullinated protein antibodies (ACPAs) [30–32]. These autoantibodies may be detected years before the onset of clinical symptoms, suggesting a role of autoantibodies in early RA development [33, 34]. Anti-ACPA-positive and anti-ACPA-negative RA can be regarded as two disease entities with different predisposing factors, etiology, disease severity, prognosis, and presumably pathogenesis [35, 36]. Furthermore, the presence of ACPA significantly worsens the course of RA, as the presence of these antibodies is associated with the rapid development of deformities, visceral lesions, and comorbidities and a high risk of death [37–39]. The presence of these autoantibodies is also associated with an increase in disease activity measured by DAS28, X-ray progression, ultrasound of the joints, and a lower probability of achieving disease remission [40, 41]. Several studies have shown that the citrullination of proteins and the synthesis of autoantibodies, which underlies the origin of RA on the mucous membranes, are under genetic regulation [31–33]. Despite the large number of studies focusing on the genetic landscape of RA, most studies focused on Caucasian and East Asian (mainly Japanese and Chinese) populations. At present, studies on the Central Asian population are scarce. Nevertheless, the study of genetic markers associated with RA among various ethnicities opens up the opportunity to identify ethnic-specific RA risk genes, which may lead to the development of ethnic-specific diagnostic and therapeutic approaches. Moreover, while genetic predisposition has been investigated for RA in general, no studies focus on genetic markers linked to ACPA-positive RA.

## 2. Materials and Methods

### 2.1. Patients and Controls

70 patients, all female, with the established RA diagnosis according to ACR/EULAR 2010 rheumatoid arthritis classification criteria [41], with the disease duration more than 1 year, were recruited in Nur-Sultan. The study was approved by the local ethics committee of National Laboratory Astana, Nazarbayev University, protocol No. 03-2019. Informed consent was obtained from all patients; the study was performed in accordance with the rules and principles of the Helsinki Declaration. All patients were examined by rheumatologists; all anamnestic and examination data were recorded in the individual cards. Examination of the bone and joint system was carried out according to generally accepted rules. The count of tender joint number (TJC) and swollen joint number (SJC) was carried out. The symptoms of transverse compression of the hands and feet and the strength of compression of the hands were evaluated. The deformities and disfigurations were recorded. Data on extra-articular manifestations and complication presence were recorded. The patient global assessment, indicated by marking a 10 cm line between very good and very bad state, was performed; the overall average score was analyzed. The assessment of disease activity was carried out according to the DAS-28 disease activity index [42]. The functional disability was assessed by the health assessment questionnaire (HAQ). The X-ray stage was set according to the X-ray images of patients over the past year.

All patients underwent laboratory examination. Blood sampling was carried out strictly after a 12–14-hour period of fasting. All patients underwent general clinical research methods with the determination of indicators of the general blood test: the content of hemoglobin, erythrocytes, platelets, leukocyte formula, and erythrocyte sedimentation rate (ESR) according to Westergren and the determination of indicators of general urinalysis. Biochemical blood tests determined the levels of alanine aminotransferase (ALT), aspartate aminotransferase (AST), total protein, protein fractions, creatinine, cholesterol, glucose, and C-reactive protein (CRP). Immunological parameters such as rheumatoid factor (RF) and antibodies to cyclic citrullinated peptide (ACPA) were also determined. The blood samples were collected from all participants in compliance with infection safety measures.

### 2.2. SNP Analysis

Genomic DNA was extracted from the collected whole blood of RA patients and healthy subjects, to the test tubes with EDTA-*ACID.* The Promega Wizard genomic DNA Purification Kit was used to DNA isolation, according to manufacturer's standard protocol. All DNA was stored at -20°C. The DNA concentration in isolated samples was determined using the NanoDrop 2000/2000c spectrophotometer (Thermo Fisher) and Qubit 2.0 using the dsDNA BR Qubit analysis kit (Thermo Fisher, catalog number 32853). The samples were genotyped for 14 SNPs (PADI2-rs761426, CD28-rs1980422, CD40-rs4810485, COG6-rs9603616, CTLA4-rs3087243, ETS1-rs73013527, FCRL3-rs2317230, LBH-rs10175798, LINC01104-rs9653442, RASGRP1-rs8032939, STAT4-rs11889341, SYNGR1-rs909685, TAGAP-rs2451258, TRAF1-rs3761847) and considered the best candidates in previous GWAS [10], by real-time polymerase chain reaction (RT-PCR) using TaqMan technology according to the manufacturer's instructions (Applied Biosystems 7500, Foster City, CA).

### 2.3. Statistical Analysis

The R ver 4.01 was used for statistical analysis: Chi square, *t*-test, and SNPAssoc package. Comparison of genotype distribution and allele frequencies between RA patients and healthy controls was evaluated by the Chi-square (*χ*2) test and Fisher's exact test, using an odds ratio (ORs) and 95% confidence intervals (95% CIs). Correlation of the associated SNP with autoantibody status among RA cases was performed with *χ*2 test. Case and control genotype frequencies did not deviate from Hardy-Weinberg equilibrium. The comparison of the clinical and laboratory parameters with the different genotypes was performed using *t*-test and *χ*2 test with Yate's correction when necessary. A *p* value lower than 0.05 was considered as statistically significant. The adjusted significance level was considered using standard borderline significance *p* value of ≤ 0.05 and Bonferroni correction.

## 3. Results

### 3.1. Demographic Description of the Study Sample and Bivariate Analysis

Sample consisted of 34 ACPA-negative and 36 ACPA-positive patients (Supplementary Table 1). The mean age of the responders is 45 (39.25-50.75) years. The highest portion of the patients presents with moderate and high activity of RA. The average age of start of the disease in patients with rheumatoid arthritis is 35.11 years. The bivariate statistical tests show the significant associations of ACPA with explanatory variables X-ray stage (*p* value = 0.017), RF status (*p* value = 0.00185), erythrocyte sedimentation rate (*p* value = 0.03218), age of RA onset (*p* value < 0.01), visual analogous scale (*p* value < 0.01), BMI (*p* value < 0.01), and gamma globulin (*p* value < 0.01).

**Figure 1:**
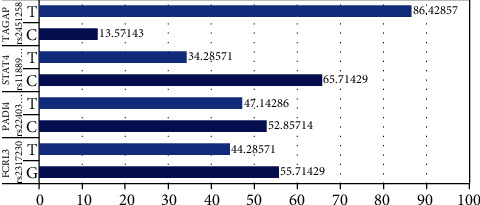
Allele frequency of significant SNPs.

Figure 1 shows the allele distribution of statistically significant single nucleotide polymorphisms. The major alleles are as follows: G (55.71%) in FCRL3 rs2317230, C (65.71%) in STAT4 rs11889341, and T (86.43%) in TAGAP rs2451258.

**Figure 2:**
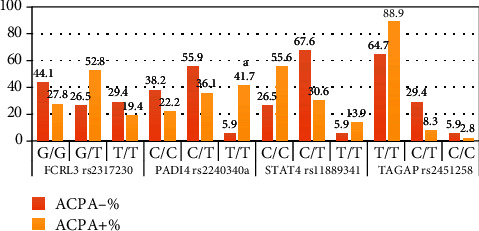
Genotype frequency of significant SNPs.

In Figure 2, the genotype distribution of statistically significant single nucleotide polymorphisms is illustrated. There is a more gradual distribution of sample in FCRL3 rs2317230 polymorphism than it is in STAT4 rs11889341 and TAGAP rs2451258 polymorphisms due to better recessive homozygous genotype distribution.

### 3.2. SNP Characteristics and Bivariate Analysis

Out of 14 SNPs of the patients tested, there was only one statistically significant result obtained using Hardy-Weinberg equilibrium (Supplementary Table 2). This can provide us with the assumption that samples are independent in 13 of the polymorphisms observed. COG6 gene SNP rs9603616 is found to violate the independence rule according to HWE and cannot be used in further analysis.

**Figure 3:**
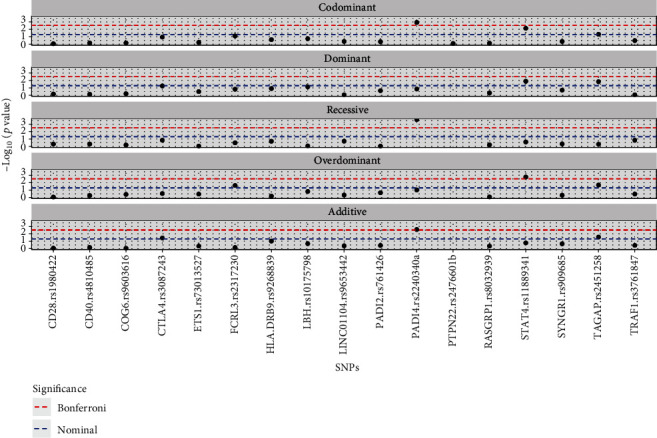
Manhattan plot of SNPs by mode of inheritance.

In Supplementary Table 2, the bivariate analysis between ACPA status and all the observed polymorphisms illustrates the statistically significant association in SNPs FCRL3 rs2317230, STAT4 rs11889341, and TAGAP rs2451258. Also, it is noticeable that polymorphism STAT4 rs11889341 is shown to have statistical significance with Bonferroni correction (Figure 3).

It is possible to observe the significance (*p* value = 0.023549316) of G/T genotype in FCRL3 rs2317230 polymorphism with an overdominant mode of inheritance with odds ratio of 3.1 (1.14-8.48) (Table 1).

Regarding STAT4, the C/T-T/T genotype in dominant mode (0.29 (0.11-0.79)) and C/T genotype in overdominant mode of inheritance (0.21 (0.08-0.58)) were significantly associated with the ACPA-negative RA form (Table 2).

Tests show significance in TAGAP rs2451258 codominant (*p* value = 0.046846672), dominant (*p* value = 0.014375636), overdominant (*p* value = 0.020765277), and log-additive (*p* value = 0.027666544) modes of inheritance (Table 3). Associations in SNP TAGAP rs2451258 are spurious due to relative small sample size and cannot be used to draw solid conclusions about its associations with ACPA, due to violation of Chi-square (*χ*2) test assumptions.

## 4. Discussion

Studies focusing on the genetic landscape of RA have contributed significantly to identifying genetic risk factors involved in disease development. Likewise, these studies also carry promise for future exploration of ethnic and racial differences in epidemiologic trends in RA, which have revealed significant variations in terms of RA incidence and prevalence worldwide. To begin to provide insight into the epidemiology of RA in Central Asia, we conducted the first pilot study to identify clinical and genetic linkages associated with ACPA positivity status in RA patients.

Consistent with data reported in previous studies on the ACPA-positive RA form, our study identified significant correlations of ACPA presence with X-ray stage, RF, ESR, age of RA onset, VAS, BMI, and Gamma globulin level and is, thus, consistent with a more severe course of the disease in our cohort of RA patients [37, 43–45]. We further found a significant association of polymorphisms in two genes, FCRL3 rs2317230 and STAT4 rs11889341, with ACPA-positive and ACPA-negative forms of RA, respectively, in Central Asian patients.

Polymorphisms of FCRL3 were previously associated with autoantibody-positive RA (RF or ACPA) in several populations and reported to independently predict radiographic progression. These findings suggest that FCRL3 is involved in both disease susceptibility and progression. Our results are partially consistent with the study by Lin et al., which identified a significant association of the FCRL3 gene with an increased RF-positive and ACPA-positive RA form in the Chinese Han population [46]. A meta-analysis of 20 previously published studies by the same authors was in accordance with the study outcome [46]. The meta-analysis also suggested that SNPs in this gene correlate with a high risk of RA development in Asian populations [46]. Partially consistent with this data, our study revealed a strong association of FCRL3 rs2317230 with the ACPA-positive form of RA, irrespective of RF status. However, researchers from Japan performed a meta-analysis of GWAS, which included 670 ACPA-negative RA patients and 16,891 controls and investigated a total of 1,948,138 markers. This study, which was followed by a replication study of the top 35 single nucleotide polymorphisms (SNPs) using 916 RA cases and 3,764 healthy subjects, showed an association between FCRL3 rs17727339 and the ACPA-negative form of RA [43]. This result is distinct from our finding showing an association with ACPA-positive status,

Our data on STAT4 association with RA are in accordance with several published studies. Meta-analyses by Tong et al. [47], Jiang et al. [48], and Ebrahimiyan et al. [49] demonstrated the association of STAT4 polymorphisms with RA susceptibility across major ethnic groups. However, the observed association in these studies was irrespective of ACPA or RF status, whereas our study revealed an association with ACPA positivity. This outcome is similar to studies in other ethnicities that reported STAT4 association with ACPA and RF status. For example, Egyptian researchers reported the association of STAT4 rs7574865 polymorphism with RF and ACPA positivity [50]. In addition, the STAT4 rs7574865 TT genotype in Syrian RA patients showed a potential impact on ACPA positivity [51]. Furthermore, a study performed by Ciccacci et al. that included 192 RA patients and 278 healthy individuals revealed STAT-4 association with a severe disease phenotype in terms of ACPA-positive status and radiographic damage in an Italian population [52].

Interestingly, there was also no significant association of polymorphisms in the PADI2 gene with the ACPA-positive form of RA in our study, despite the known key role of PADI2 and other PADI gene family members in the process of protein citrullination during RA development. Accordingly, a recent study by Guzmán-Guzmán et al. showed that PADI2 rs1005753 was associated with ACPA positivity and RF positivity in Mexican patients with RA [24]. Our study specifically investigating PADI2 rs761426 SNP association with ACPA-positive RA form and did not find any correlation. This result may thus indicate that various SNPs of one gene differ in their association with ACPA status in RA patients of diverse populations.

Recently, we published a study on HLA-DRB9, PADI4, and PTPN22 gene distribution in Kazakhstani RA patients, which revealed, among other data, an association of PADI4 with the ACPA-positive form of RA [29]. Similar to what was found in this study, PTPN22 did not show any association with RA, RF-positive, or ACPA-positive status [29].

The SNPs STAT4, CTLA4, CD40, LBH, ETS1, and TAGAP, identified earlier in Caucasian and Asian populations, as well as in African-Americans [8–10, 53], were not replicated in our study. This result is in part consistent with a study in a Latin American population [54].

## 5. Conclusion

Our study demonstrates a significant correlation of FCRL3 with the ACPA-positive form of RA, whereas STAT4 showed a significant association with the ACPA-negative form. In contrast, TAGAP SNP association with ACPA status was spurious due to violation of Chi-square (*χ*2) test assumptions. Furthermore, 11 earlier identified SNPs as being significantly associated with ACPA status in Caucasian and East Asian populations were not replicated in our cohort. We recognize that the small sample size may be a major limitation of our study. A larger cohort of RA patients and an expanded number of SNPs will be needed to confirm our findings with higher confidence levels and stronger statistical power.

Nevertheless, our current results do not fully correlate with published studies conducted on Asian and European populations, which shows the need for further research into the epidemiology of RA.

## Figures and Tables

**Table 1:** Statistically significant SNP FCRL3 rs2317230 characteristics and bivariate statistics.

FCRL3 rs2317230	ACPA-	%	ACPA+	%	OR	Lower	Upper	*p* value	AIC
Codominant									
G/G	15	44.1	10	27.8	1			0.07679	97.9
G/T	9	26.5	19	52.8	3.17	1.03	9.77		
T/T	10	29.4	7	19.4	1.05	0.3	3.68		
Dominant									
G/G	15	44.1	10	27.8	1			0.15291	98.9
G/T-T/T	19	55.9	26	72.2	2.05	0.76	5.55		
Recessive									
G/G-G/T	24	70.6	29	80.6	1			0.33037	100
T/T	10	29.4	7	19.4	0.58	0.19	1.75		
Overdominant									
G/G-T/T	25	73.5	17	47.2	1			0.02355^∗^	95.9
G/T	9	26.5	19	52.8	3.1	1.14	8.48		
Log-additive									
0,1,2	34	48.6	36	51.4	1.11	0.6	2.06	0.72789	100.9

^∗^ refers to statistically significant *p* value < 0.05 (no adjustment). ^∗∗^ refers to statistically significant *p* value < 0.01 (no adjustment).

**Table 2:** Statistically significant SNP STAT4 rs11889341 characteristics and bivariate statistics.

STAT4 rs11889341	ACPA-	%	ACPA+	%	OR	Lower	Upper	*p* value	AIC
Codominant									
C/C	9	26.5	20	55.6	1			0.00716	93.1
C/T	23	67.6	11	30.6	0.22	0.07	0.62		
T/T	2	5.9	5	13.9	1.12	0.18	6.93		
Dominant									
C/C	9	26.5	20	55.6	1			0.01268^∗^	94.8
C/T-T/T	25	73.5	16	44.4	0.29	0.11	0.79		
Recessive									
C/C-C/T	32	94.1	31	86.1	1			0.25659	99.7
T/T	2	5.9	5	13.9	2.58	0.47	14.31		
Overdominant									
C/C-T/T	11	32.4	25	69.4	1			0.00169^∗^	91.1
C/T	23	67.6	11	30.6	0.21	0.08	0.58		
Log-additive									
0,1,2	34	48.6	36	51.4	0.6	0.28	1.26	0.16957	99.1

^∗^ refers to statistically significant *p* value < 0.05 (no adjustment). ^∗∗^ refers to statistically significant *p* value < 0.01 (no adjustment).

**Table 3:** Statistically significant SNP TAGAP rs2451258 characteristics and bivariate statistics.

TAGAP rs2451258	ACPA-	%	ACPA+	%	OR	Lower	Upper	*p* value	AIC
Codominant									
T/T	22	64.7	32	88.9	1			0.04685	96.9
C/T	10	29.4	3	8.3	0.21	0.05	0.84		
C/C	2	5.9	1	2.8	0.34	0.03	4.03		
Dominant									
T/T	22	64.7	32	88.9	1			0.01437^∗^	95
C/T-C/C	12	35.3	4	11.1	0.23	0.07	0.8		
Recessive									
T/T-C/T	32	94.1	35	97.2	1			0.51842	100.6
C/C	2	5.9	1	2.8	0.46	0.04	5.29		
Overdominant									
T/T-C/C	24	70.6	33	91.7	1			0.02076^∗^	95.6
C/T	10	29.4	3	8.3	0.22	0.05	0.88		
Log-additive									
0,1,2	34	48.6	36	51.4	0.34	0.12	0.97	0.02766^∗^	96.1

^∗^ refers to statistically significant *p* value < 0.05 (no adjustment). ^∗∗^ refers to statistically significant *p* value < 0.01 (no adjustment).

## Data Availability

Data and material used during this study will be available from the corresponding author upon request.

## Additional Points

*Study Limitation*. We recognize that the small sample size may be a major limitation of our study. A larger cohort of RA patients, control group, and an expanded number of SNPs will be needed to confirm our findings with higher confidence levels and stronger statistical power.

## Supplementary Materials

Supplementary 1 Supplementary Table 1: description of clinical characteristics and bivariate analysis.

Supplementary 2 Supplementary Table 2: SNP descriptive statistics and bivariate analysis.
