# Supplementary material for: Association Study of Anticitrullinated Peptide Antibody Status with Clinical Manifestations and SNPs in Patients Affected with Rheumatoid Arthritis: A Pilot Study
Source: Dis Markers. 2022 May 11;2022:2744762. doi: 10.1155/2022/2744762 (PMC9118096; doi:10.1155/2022/2744762)
Supplement: Supplementary 1 — Supplementary Table 1: description of clinical characteristics and bivariate analysis. [file 2744762.f1.docx]

Supplementary Table 1. Description of clinical characteristics and bivariate analysis

| Characteristic | Description | P-value |
| --- | --- | --- |
| ACPA  ACPA-  ACPA+ | 34  36 | - |
| Age | 45.07 (39.25 - 50.75) | 0.6717 |
| DAS28 | 4.823 (3.828 - 6.145) | 0.2761 |
| Activity  High  Low  Moderate  Remission | 26  8  32  4 | 0.3166 |
| Functional class  Class I  Class II  Class III  Class IV | 19  42  8  1 | 0.2254 |
| X-ray stage  Stage I  Stage II  Stage III  Stage IV | 15  29  16  10 | 0.01701* |
| RF  RF-  RF+ | 17  53 | 0.00185** |
| HAQ | 0.9877 (0.4100 - 1.6300) | 0.414 |
| ESR (mm.h) | 30.49 (14.25 - 46.00) | 0.03218* |
| Heredity for RA  No  Yes | 48  22 | 0.5413 |
| Age of RA onset | 35.11 (26.00 - 42.75) | <0.01** |
| VAS | 4.739 (3.000 - 6.000) | <0.01** |
| BMI | 25.27 (21.60 - 28.60) | <0.01** |
| CMV  No  Yes | 62  8 | 1 |
| Herpes  No  Yes | 54  16 | 0.469 |
| TBS  No  Yes | 66  4 | 1 |
| Hepatitis À  No  Yes | 58  12 | 0.8348 |
| Measles  No  Yes | 64  6 | 0.1755 |
| Rubella  No  Yes | 65  5 | 0.3198 |
| Chicken pox  No  Yes | 41  29 | 1 |
| Sex infection  No  Yes | 49  21 | 1 |
| Oncology  No  Yes | 69  1 | 1 |
| Trauma  No  Yes | 48  22 | 0.9238 |
| Alcohol  No  Yes | 68  2 | 1 |
| Allergy  No  Yes | 40  30 | 1 |
| Education  Higher  Secondary | 38  32 | 0.9836 |
| Social  No  Yes | 27  43 | 0.07579 |
| Professional harm  No  Yes | 62  8 | 1 |
| Anemia  No  Yes | 42  28 | 0.5913 |
| Hypergammaglobuliemia  No  Yes | 29  41 | 0.8406 |
| Gamma globulin | 20.17 (17.62 - 22.07) | <0.01* |
| Breastfeeding | 14.7 (9.0 - 20.0) | 0.3328 |
| BF.24  No  Yes  NA's | 63  6  1 | 1 |
|  |  |  |

* - refers to statistically significant p-value<0.05 (no adjustment)

** - refers to statistically significant p-value<0.01 (no adjustment)
