# Supplementary material for: Association Study of Anticitrullinated Peptide Antibody Status with Clinical Manifestations and SNPs in Patients Affected with Rheumatoid Arthritis: A Pilot Study
Source: Dis Markers. 2022 May 11;2022:2744762. doi: 10.1155/2022/2744762 (PMC9118096; doi:10.1155/2022/2744762)
Supplement: Supplementary 2 — Supplementary Table 2: SNP descriptive statistics and bivariate analysis. [file 2744762.f2.docx]

Supplementary table 2. SNPs descriptive statistics and bivariate analysis

| SNPs | Alleles | Major allele frequency | HWE | Mode of inheritance | | | | Log-additive |
| --- | --- | --- | --- | --- | --- | --- | --- | --- |
|  |  |  |  | Codominant | Dominant | Recessive | Overdominant |  |
| CD28  rs1980422 | T/C | 87.1 | 1 | 0.885132886 | 0.678685796 | 0.485714286 | 0.896443613 | 0.885132886 |
| CD40  rs4810485 | G/T | 82.1 | 0.679617 | 0.702310102 | 0.740475141 | 0.485714286 | 0.550429055 | 0.702310102 |
| COG6  rs9603616 | C/T | 68.6 | 0.026734* | 0.672923217 | 0.62205927 | 0.665852253 | 0.384099311 | 0.904457388 |
| CTLA4  rs3087243 | G/A | 69.3 | 0.410964 | 0.112106048 | 0.05464699 | 0.14726357 | 0.297890502 | 0.036483448 |
| ETS1  rs73013527 | C/T | 65 | 0.440043 | 0.587506717 | 0.32125462 | 0.922239252 | 0.35233583 | 0.454469569 |
| FCRL3  rs2317230 | G/T | 55.7 | 0.144555 | 0.076791119 | 0.15291301 | 0.330370746 | 0.023549316* | 0.727887574 |
| LBH  rs10175798 | A/G | 52.1 | 0.810475 | 0.18445553 | 0.072863335 | 0.867701885 | 0.153644399 | 0.214477559 |
| LINC01104  rs9653442 | T/C | 60 | 0.625773 | 0.427484334 | 0.862869005 | 0.198254562 | 0.468421337 | 0.429808516 |
| PADI2  rs761426 | T/A | 67.1 | 0.587174 | 0.462512575 | 0.239900246 | 0.941639888 | 0.228110958 | 0.372470726 |
| RASGRP1  rs8032939 | C/T | 51.4 | 0.337624 | 0.748173653 | 0.483895026 | 0.631862531 | 0.83593051 | 0.457542368 |
| STAT4  rs11889341 | C/T | 65.7 | 0.60392 | 0.007162581* | 0.012679042* | 0.256599824 | 0.001687741* | 0.169570496 |
| SYNGR1  rs909685 | A/T | 60 | 0.211093 | 0.434251769 | 0.203075198 | 0.472767065 | 0.494204849 | 0.223311995 |
| TAGAP  rs2451258 | T/C | 86.4 | 0.099496 | 0.046846672* | 0.014375636* | 0.518427403 | 0.020765277* | 0.027666544* |
| TRAF1  rs3761847 | A/G | 57.9 | 0.807904 | 0.334624219 | 0.862869005 | 0.14979298 | 0.344476375 | 0.369657619 |

* - refers to statistically significant p-value<0.05 (no adjustment)

** - refers to statistically significant p-value<0.01 (no adjustment)
